# Synergistic effect of Maquiberry cystatin, sodium fluoride and stannous chloride for the prevention of initial dental erosion *in vitro*

**DOI:** 10.1590/1678-7757-2024-0479

**Published:** 2025-05-23

**Authors:** Vinícius Taioqui PELÁ, Thelma Lopes SILVA, Even Akemi TAIRA, Geórgia Almeida SANT’ANA, Letícia Oba SAKAE, Flávia Mauad LEVY, Taís Scaramucci FORLIN, Eduardo Pereira de SOUZA, Talita Mendes Oliveira VENTURA, Thiago Saads CARVALHO, Adrian LUSSI, Marília Afonso Rabelo BUZALAF

**Affiliations:** 1 Universidade de São Paulo Faculdade de Odontologia de Bauru Departamento de Ciências Biológicas Bauru SP Brasil Universidade de São Paulo, Faculdade de Odontologia de Bauru, Departamento de Ciências Biológicas, Bauru, SP, Brasil.; 2 Universidade de São Paulo Faculdade de Odontologia Departamento de Odontologia Restauradora São Paulo SP Brasil Universidade de São Paulo, Faculdade de Odontologia, Departamento de Odontologia Restauradora, São Paulo, SP, Brasil.; 3 Universidade Federal de São Carlos Departamento de Genética e Evolução São Carlos SP Brasil Universidade Federal de São Carlos, Departamento de Genética e Evolução, São Carlos, SP, Brasil.; 4 University of Bern School of Dental Medicine Department of Restorative Bern Switzerland University of Bern, School of Dental Medicine, Department of Restorative, Preventive and Pediatric Dentistry, Bern, Switzerland.; 5 University Hospital for Conservative Dentistry and Periodontology Medical University of Innsbruck Innsbruck Austria University Hospital for Conservative Dentistry and Periodontology, Medical University of Innsbruck, Innsbruck, Austria and Department of Restorative, Preventive and Pediatric Dentistry, School of Dental Medicine, Bern, Switzerland.

**Keywords:** Demineralization, Enamel, Saliva

## Abstract

**Objective:**

This study analyzed the synergistic effect between a recombinant Maquiberry (MaquiCPI-3) protein, sodium fluoride (NaF), and stannous chloride (SnCl2) against initial dental erosion *in vitro*.

**Methodology:**

A total of 98 bovine enamel samples were prepared and allocated to seven treatment groups (n=14/group) as follows: deionized water (Water); commercial solution, 800 ppm Sn^+2^, 500 ppm F-, Elmex^TM^, Erosion, GABA, Therwil, BL, CH (Elmex); 500 ppm of fluoride (F^-^) from NaF (NaF); 500 ppm of F^-^ from NaF and 800 ppm of tin (Sn^+2^) from SnCl_2_ (NaF+SnCl); 0.5 mg/mL MaquiCPI-3 (MaquiCPI-3); combination of MaquiCPI-3 and NaF (Maqui+NaF) and; combination of MaquiCPI-3, NaF and SnCl (Maqui+NaF+SnCl). Samples were treated with the respective solutions (250 μl, 2 min, 37°C, 250 rpm). After forming the acquired enamel pellicle (AEP) by adding human saliva from nine volunteers (250 μl, 1 h, 37°C, 250 rpm), the samples underwent acid challenge (1 mL, 1% citric acid, pH 3.6, 1 min, 25°C, 250 rpm). All procedures were performed in triplicate. Surface microhardness change percentage (%SMC) and relative surface reflection intensity (%SRI) were measured and analyzed by One-way ANOVA/Tukey’s tests (p<0.05).

**Results:**

The Elmex, NaF, NaF+SnCl, MaquiCPI-3, Maqui+NaF, and Maqui+NaF+SnCl groups showed significantly lower %SMC compared with Water. The NaF, Sn^+2^, NaF+SnCl, MaquiCPI-3, Maqui+NaF and Maqui+NaF+SnCl groups exhibited significantly greater protection compared with Elmex. Maqui+NaF+SnCl revealed better enamel protection (significant) when compared with the MaquiCPI-3 and Maqui+NaF groups. Elmex, NaF+SnCl, MaquiCPI-3, Maqui+NaF, and Maqui+NaF+SnCl had a significantly higher %SRI compared to the Water and NaF groups, which did not differ between each other.

**Conclusion:**

All treatment solutions provided protection against initial dental erosion *in vitro*. Formulations containing Maqui+NaF+SnCl_2_ offered superior enamel protection compared with MaquiCPI-3 alone.

## Introduction

Initial dental erosion is characterized by the demineralization of tooth tissues, resulting from exposure to non-bacterial acids.^1^ This chemically induced process leads to tooth surface softening thereby making it more susceptible to subsequent abrasive forces, a condition known as erosive tooth wear (ETW).^1,2^ Interactions between erosive and abrasive processes can intensify the magnitude of the wear, promoting the progressive enamel removal and potentially affecting the dentin structure.^3,4^ This is of particular concern given the high ETW prevalence among children and adolescents.^5,6^ Dietary factors are the main contributors for this condition, especially the frequent consumption of acidic foods and beverages.^7^ Moreover, ETW prevalence increases with age, highlighting its progressive nature across the aging process.^8^

Clinically, early detection of ETW is crucial for its effective management through interventions encompassing both treatment and preventive measures.^9^ The latter strategies include dietary modifications, reduced exposure to acidic substances, and the use of dental products designed to control the condition.^10^ Nevertheless, the availability of products targeting specifically ETW remains limited. One mouth rinse commercially available which contains inorganic components, specifically F^-^ and Sn^+2^,^11^ has shown efficacy in controlling erosion in its initial stages^12,13^ and ETW.^14,15^ The synergism between these inorganic components can protect the enamel against demineralization and decrease its solubility.^12,16-18^Despite the well-documented protective effects of inorganic components against dental erosion, commercially available formulations containing this combination are not widely accessible worldwide. Additionally, these formulations present challenges related to stability and user acceptance, which may limit their clinical applicability.^15^

In the field of dental product development, an innovative approach has emerged for ETW prevention through use of organic components,^19^ particularly recombinant proteins.^20^ These proteins are applied to the enamel surface following the acquired pellicle engineering procedure to strengthen this integument with acid-resistant proteins and thus enhance the preventive effect against dental erosion.^21^ A sugarcane cystatin (CaneCPI-5) has shown efficacy in preventing initial dental erosion and ETW across various experimental approaches, including *in vitro*^12,20,22^ and clinical studies.^13,15,23^ Subsequently, a study conducted the transcriptomic analysis of Maquiberry leaves, a perennial plant native to South America [*Aristotelia chilensis (Mol.) Stuntz*],^24^ and cloned a novel cystatin, designated as MaquiCPI-3. In a previous investigation, MaquiCPI-3 showed a comparative advantage over CaneCPI-5 due to its ability to offer superior enamel protection against initial erosive challenges, in addition to its higher production yield through recombinant techniques.^24^

Acknowledging the novelty of studies utilizing organic components and the established effectiveness of inorganic components through commercial dental products, both aimed at controlling ETW,^11,13,15^ another avenue emerges by combining these active principles to enhance the protective effect against erosive challenges.^12^ CaneCPI-5 combined with sodium fluoride (NaF) has shown significant protection against initial dental erosion *in vitro* when compared with solutions containing only isolated components.^12^ This enhancing strategy has also been employed in studies involving the combination of casein and F^-25^ plant extracts and F^-16^ and proanthocyanidin and vitamin E^26^ for enamel, as well as the combination of collagen and F^-27^ and polyphenols and F^-28^for dentin.

As such, it becomes relevant to test a solution combining MaquiCPI-3, NaF, and SnCl_2_ as potential agents for an improved protection against initial dental erosion. By harnessing the benefits of these components, the *in vitro* study represents a significant first step toward more effective strategies to control ETW. In this regard, MaquiCPI-3 addition may offer increased protection by interacting with the acquired enamel pellicle (AEP), reinforcing this protective layer with other acid-resistant proteins. Thus, combining organic and inorganic components enhances the protective mechanism of enamel, either directly on the enamel surface (as in the case of F^-^ and Sn^2^ ions) or indirectly through AEP fortification (as facilitated by MaquiCPI-3).

In light of the above, there is a need to explore alternative strategies that not only enhance enamel protection but also improve formulation stability and patient acceptability. MaquiCPI-3 combined with inorganic components presents a promising approach, as this protein may complement the effects of fluoride and stannous chloride by providing an additional layer of protection against acid challenges. However, its efficacy in combination with NaF and SnCl₂ remains underexplored, particularly regarding its ability to enhance their preventive effects through synergistic mechanisms. Investigating this combination could lead to the development of more effective formulations for dental erosion prevention, addressing the current limitations of commercially available solutions. Further research is therefore necessary to determine whether the combination of MaquiCPI-3, NaF, and SnCl₂ offers a novel and potentially more effective approach to preventing ETW. This innovative approach not only shows the continuous evolution in the development of dental products but also underscores the importance of exploring these synergies to achieve optimized therapeutic outcomes for ETW control, thereby contributing to improve oral health.

Thus, this study analyzed the synergistic effect of MaquiCPI-3 combined with NaF and SnCl_2_ to enhance the preventive effect against initial dental erosion *in vitro*.

The following null hypotheses were formulated:

1. MaquiCPI-3 does not prevent initial dental erosion *in vitro*;
2. NaF does not enhance the preventive effect of MaquiCPI-3 against initial dental erosion *in vitro*;
3. The combination of NaF and SnCl_2_ does not have a synergistic effect to enhance the prevention of initial dental erosion *in vitro*;
4. The combination of MaquiCPI-3, NaF and SnCl_2_ does not have a synergistic effect to enhance the prevention of initial dental erosion *in vitro* compared with MaquiCPI-3 isolated.

## Methodology

### Ethical considerations

The study was reviewed and approved by the Research Ethics Committee of the Bauru School of Dentistry, University of São Paulo (75831123.4.0000.5417). This approval covered both the involvement of human subjects (collection of stimulated saliva) and animal use (acquisition of bovine teeth samples; 012/2023). To ensure ethical standards, all human participants received with detailed information about the study’s purpose, procedures, potential risks, and benefits. This agreement was formalized by signing an Informed Consent Form.

### Acquisition and preparation of bovine teeth

Freshly extracted bovine teeth were properly preserved in a 0.1% thymol solution (Sigma–Aldrich Co., St. Louis, MO, USA) with the pH adjusted to 7.0 and kept at room temperature. Before proceeding with the analyses, the teeth underwent a visual inspection to identify possible stains and cracks; those presenting such imperfections were excluded from the study. After removing the roots of the selected teeth using a specific cutting device (Maruto, Tokyo, JPN), the dental crowns were mounted and fixed with thermoplastic impression compound (Kerr Corporation, Orange, CA, EUA) onto acrylic plates measuring 40x40x5 mm^3^. These plates were carefully positioned and fixed on the ISOMET Low Speed Saw (Buehler, Lake Bluff, IL, USA), a precision cutting device. During the cutting procedure, two double-sided diamond discs model XL 12205, with “high concentration” specifications and dimensions of 12.0x0.3x12.7 mm^3^ (Extec Corp., Enfield, CT, USA), were used. Cutting was performed at 300 rpm and constantly cooled with deionized water, ensuring adequate precision and control throughout the procedure. A total of 98 enamel samples were obtained with standardized dimensions of 4 x 4 mm^2^.^20^

### Standardization of polishing process

Each sample was affixed at the center of an acrylic disc forming a disc/tooth ensemble, which was then placed onto a Metallographic Polisher (Buehler, Lake Bluff, Il, USA). Within this equipment, dentin planification was performed using a silicon carbide sandpaper with a 600 grit size (Carborundum discs of Al_2_O_3_ papers; Buehler, Lake Bluff, IL, USA) under deionized water cooling. The procedure was repeated until the specimens reached a thickness of approximately 3 mm. Next, the samples were removed from the acrylic discs and reattached at the center of an acrylic plate with the enamel surface facing upward to continue the polishing process. The ensemble was then adapted to the polisher, where the enamel surface was abraded with a silicon carbide sandpaper with a 600 grit size (Carborundum discs of Al_2_O_3_ papers; Buehler, Lake Bluff, IL, USA) under water cooling for 2 min. Subsequently, polishing was performed using a silicon carbide sandpaper with a 1200 grit size (Carborundum discs of Al_2_O_3_ papers; Buehler, Lake Bluff, IL, USA), also under water cooling, for 2 min to remove approximately a total of 130 μm from the enamel surface.

Polishing was concluded with a felt pad moistened with a 1 μm diamond suspension (felt papers: Buehler, Lake Bluff, IL, USA; water-based diamond polishing suspension: Extec, IL, USA) applied for 3 min at high speed. To prevent interference from the grains of the previous sandpapers on the subsequent polishing quality, the disc/tooth ensemble was washed with deionized water after each step and at the end of polishing using an ultrasonic device (T7 Thornton, operating at a frequency of 40 KHz, São Paulo, SP, BR) for 3 min.^29^

### Participant selection and stimulated saliva collection

A total of nine participants of both sexes (5 women and 4 men) and an average age of 30 years were selected for the study. To ensure representativeness and minimize potential variables, participants were required to have good general health, excluding individuals who met the following criteria: smokers, pregnant women, patients with ongoing systemic diseases, and those regularly using medication. Participants were also required to have good oral health, with individuals excluded if they presented with active dental caries, ETW, or periodontal disease.^15^ Salivary flow assessment was another important aspect in participant selection. Volunteers were considered eligible if they presented with salivary flow within established normal parameters, i.e., greater than 0.3 mL/min for unstimulated saliva and greater than 1.0 mL/min for stimulated saliva.^15^

One week before collection, participants were provided with oral hygiene kits containing a toothbrush, toothpaste, and dental floss (Oral B^®^, The Procter & Gamble Company, Cincinnati, OH, USA) to standardize oral hygiene conditions for all individuals. Prior to saliva collection, participants were instructed to abstain from consuming food and beverages (except water) for 2 h. Collection of stimulated whole saliva was performed in 50 mL tubes for 10 min using parafilm (Sigma-Aldrich, St. Louis, MO, USA), in an environment with temperature controlled by ice, between 9 and 10 a.m. Subsequently, saliva samples were centrifuged at 14,000 g for 20 min at 4°C (Corning Inc, Corning, NY, USA). The supernatants were collected to form a saliva pool, and aliquots were then made and stored at -80°C until the experiment.^22^

### Production of the new maquiberry-derived protein (MaquiCPI-3)

MaquiCPI-3 heterologous expression used the bacterial strain *E. coli* Rosetta (DE3) transformed with plasmid, as previously described.^24^ Protein production was initiated from the induced bacterial culture by adding IPTG (Isopropyl-beta-D-Thiogalactoside, Sigma–Aldrich Co., St. Louis, MO, USA). Subsequently, the expressed protein underwent centrifugation and sonication protocol. Affinity chromatography was used to purify the MaquiCPI-3 protein. In this process, columns containing Ni-NTA Superflow resin (Thermo Fisher Scientific, Waltham, MA, USA) were used, following protocols established in previous studies.^20,24^

### Division of experimental groups

The 98 samples were randomized and allocated into seven treatment groups (n=14/group) as follows:

1. Deionized water, pH 7.00 (Negative control; Water);
2. Commercial solution, 800 ppm Sn^2^, 500 ppm F^-^, Elmex^TM^, pH 4.43 – Erosion Protection, GABA, Therwil, BL, CH) (Positive control; Elmex);^13^
3. 0.5 mg/mL MaquiCPI-3, pH 7.30 (MaquiCPI-3);^24^
4. 500 ppm of F^-^ from NaF, pH 4.50, Sigma–Aldrich Co., St. Louis, MO, USA (NaF);^12^
5. 500 ppm of F^-^ from NaF and 800 ppm of Sn^2^ from SnCl_2_, pH 4.50, Sigma–Aldrich Co., St. Louis, MO, USA (NaF+SnCl);
6. Combination of 0.5 mg/mL MaquiCPI-3 and 500 ppm F^-^, pH 7.45 (Maqui+NaF);
7. Combination of 0.5 mg/mL MaquiCPI-3, 500 ppm of F^-^ and 800 ppm Sn^2^, pH 7.33 (Maqui+NaF+SnCl).

Control groups were used as originally produced, without any manipulation; all other treatments were directly solubilized in deionized water. The group containing Sn^2^⁺ was stabilized with D-gluconic acid (0.23%) to prevent potential precipitation of this inorganic component. Thus, the solution was prepared in the following order: first, D-gluconic acid (0.23%) was added to deionized water, followed by 800 ppm of Sn^2^⁺ from SnCl₂, and finally, 500 ppm of F⁻ from NaF. The resulting native pH of 3.89 was adjusted to 4.5. For the groups containing MaquiCPI-3, the lyophilized protein was added last and the native pH was maintained, reflecting the natural pH of the preparation. The pH of the solutions was determined using a calibrated pH meter before each experimental phase to ensure accuracy and consistency.

### Treatment, acquired pellicle formation, and acid challenge

**Figure 1:**
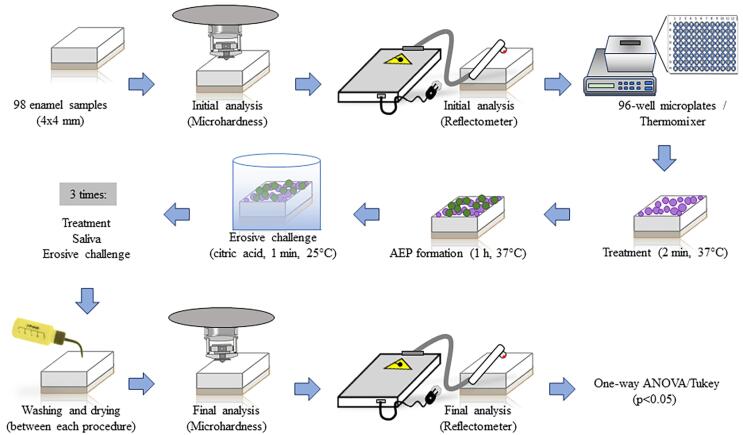
Steps of the experimental procedures. Once the 98 samples of bovine enamel were prepared, they underwent initial microhardness and reflectometer analyses. Subsequently, they were individually incubated in different treatments (Water, Elmex, NaF, NaF+SnCl, MaquiCPI-3, Maqui+NaF, and Maqui+NaF+SnCl) for 1 min. Next, the acquired enamel pellicle was formed for 1h. The erosive process was individually performed by incubating the samples in 1 mL of 1% citric acid for 1 min. All experimental procedures were performed in triplicate, and all samples were washed and dried with compressed air between each procedure. After the final microhardness and reflectometer analyses, the data were analyzed by ANOVA/Tukey’s tests (p<0.05).

Each sample was individually treated with 250 μl of the respective treatment for 2 min at 37 °C under constant agitation (250 rpm) (Thermo Fisher Scientific, Waltham, MA, USA).^12^ Subsequently, AEP was formed by adding 250 μl of stimulated human saliva for 1 hour at 37 °C, also under constant agitation.^30^ After AEP formation, the samples underwent an acid challenge in which they were immersed in 1 mL of 1% citric acid solution, pH 3.6, for 1 min at 25 °C, under the same agitation.^20^ The native pH of the acid solution of 2.8 was adjusted to 3.5 to follow the protocols for initial dental erosion.^12^ Between each experiment stage (treatment, AEP formation, and acid challenge), the samples were carefully rinsed for 5 s and dried for 3 s with compressed air, ensuring the removal of residues and maintaining controlled experimental conditions. These procedures were repeated once daily for three consecutive days, using 96-well acrylic plates (Sigma-Aldrich, St. Louis, MO, USA) to organize and handle the samples.^24^ During intervals between procedures and overnight, the samples were stored in humidity chambers at 4 °C, ensuring the preservation of experimental conditions until the next session^12^(Figure 1).

### Surface microhardness analysis

Surface microhardness (SM) of the enamel was assessed using a Knoop indenter with a load of 50 g for 15 s on the Surface Microhardness Tester (AMS – HMV-2000; Shimadzu, Kyoto, JPN). This analysis was performed at two distinct times: before the experiment (SM_baseline_) and after the experimental period (SM_final_). During measurements, five indentations were made at specific locations on the left side of each sample, with a spacing of 25 µm between them. Control indentations were made using loads of 2 g and 5 g, ensuring safety against possible surface loss. Percentage of surface microhardness change (%SMC) was calculated from the baseline and final surface microhardness measurements using the following equation: %SMC = ([SM_baseline_ – SM_final_]/SM_baseline_) × 100.^20^

### Surface reflection analysis

Surface reflection intensity (SRI) analysis was conducted using a portable reflectometer (Optipen, University of Bern, Bern, BE, Switzerland) before (SRI_baseline_) and after the experimental period (SRI_final_).^13,22,23,31^ First, each sample was carefully dried with compressed air and the tip of the reflectometer was positioned directly on the enamel surface. Then, the device tip was tilted at different angles until reaching the highest SRI point, which was recorded through specific software operated on a notebook connected to the reflectometer. This analysis was performed in an area adjacent to the indentations used for microhardness measurements. Generally, higher SRI values indicated a smoother enamel surface, suggesting a lower degree of erosion. Conversely, lower SRI values were associated with rougher enamel surfaces which may indicate higher erosion. For statistical analysis, the relative SRI value (%SRI) was calculated using the following equation: %SRI = (SRI_final_/SRI_baseline_) × 100.

### Statistical analysis

Statistical analysis was performed using GraphPad Prism software (Version 6.0 for Windows, GraphPad Software Inc., La Jolla, CA, USA). Initially, the data underwent an evaluation regarding the normality of distribution using the Kolmogorov-Smirnov test. A homogeneity test was conducted using the Bartlett test to verify if the group variances were equal. %SMC and %SRI data were analyzed by one-way ANOVA and Tukey’s Multiple Comparison Test. Significance level was set at 5%, meaning that differences between groups were considered statistically significant when p-value < 0.05.

## Results

### Surface microhardness analysis

**Figure 2:**
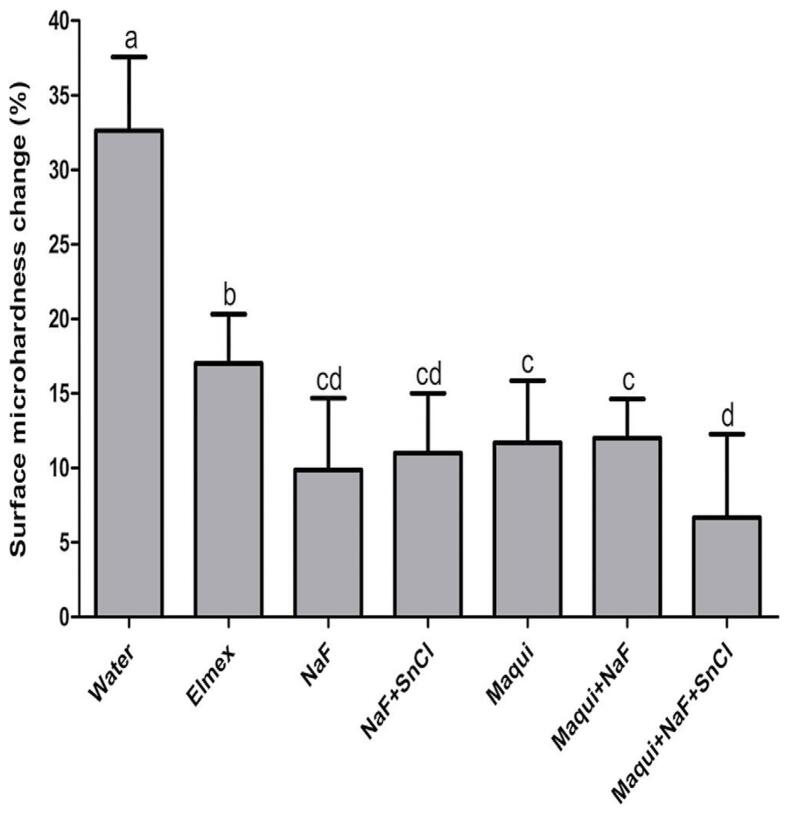
Surface microhardness changes percentage (%SMC) according to different solutions: Water, Elmex, NaF, NaF+SnCl, MaquiCPI-3, Maqui+NaF, and Maqui+NaF+SnCl. Smaller bars indicate less erosion. Error bars represent the standard deviation. Different letters mean significant differences between treatments (ANOVA followed by Tukey’s tests, p<0.05, n=14).

%SMC results (mean/standard deviation) indicated that the Elmex (17.01/3.30), NaF (9.87/4.79), NaF+SnCl (11.00/4.00), MaquiCPI-3 (11.68/4.17), Maqui+NaF (11.98/2.64), and Maqui+NaF+SnCl (6.67/5.58) groups exhibited significantly lower (F: 58.80) surface microhardness changes (indicating enamel protection) compared with the Water group (32.63/4.91). Moreover, the NaF, NaF+SnCl, MaquiCPI-3, Maqui+NaF, and Maqui+NaF+SnCl groups showed similar and significantly higher enamel protection compared with Elmex. Additionally, the group treated with Maqui+NaF+SnCl had the best enamel protection (significant) when compared with MaquiCPI-3 and Maqui+NaF (p<0.05) (Figure 2).

### Surface reflection analysis

**Figure 3:**
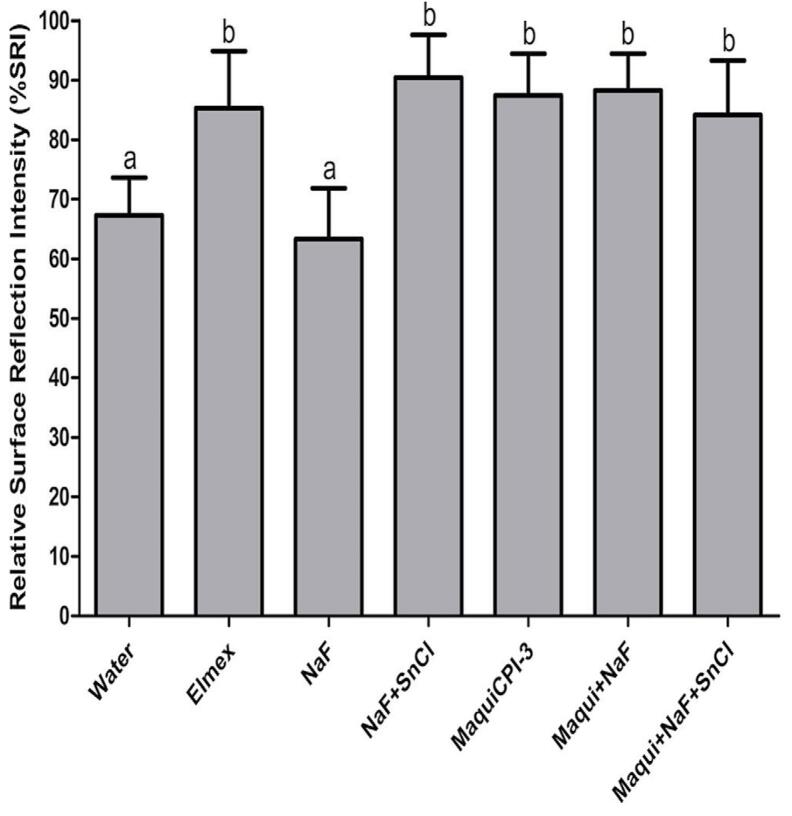
Relative surface reflection intensity (%SRI) according to different solutions: Water, Elmex, NaF, NaF+SnCl, MaquiCPI-3, Maqui+NaF, and Maqui+NaF+SnCl. Higher bars indicate less erosion. Error bars represent the standard deviation. Different letters mean significant differences between treatments (ANOVA followed by Tukey’s tests, p<0.05, n=14).

%SRI results (mean/standard deviation) indicated that the Elmex (85.33/9.54), NaF+SnCl (90.43/7.16), MaquiCPI-3 (87.42/6.99), Maqui+NaF (88.24/6.19), and Maqui+NaF+SnCl (84.16/9.17) groups exhibited a significantly higher (F: 30.28) %SRI (indicating enamel protection) compared with the Water (67.33/6.31) and NaF (63.31/8.56) groups, which did not differ from each other (p<0.05) (Figure 3).

## Discussion

This study focused on the use of a novel treatment solution comprising both organic and inorganic components to prevent initial dental erosion *in vitro*. The synergistic effect between these active principles was a crucial strategy to achieve enhanced enamel protection for ETW control in future clinical applications. As this innovative approach is in its initial phase, it is imperative to design the study using an *in vitro* protocol. This method allows for preliminary screening, which is crucial for identifying the most promising treatments to be tested further in studies with greater clinical relevance.^32^

To achieve this, the methodological aspects implemented should be considered when interpreting the results, such as the use of bovine enamel samples, given the lack of significant difference between bovine and human enamel in protocols that involve AEP formation.^33^ Regarding the erosion protocols, it is important to note that bovine enamel may be used according to established recommendations for erosion studies, as it is widely accepted in the literature. Extending the duration could lead to advanced enamel degradation, compromising microhardness measurements. This makes bovine enamel a suitable alternative in experimental protocols, particularly when human enamel is not available or practical for use.^34^

Treatment application time was set at 2 min,^30^ aligning it more closely with the 1 min duration typical of mouth rinse protocols^21^ and in accordance with previous similar methodologies used by our group.^12^ Treatment application to the enamel surface was done prior to AEP formation due to “AEP engineering,” a novel approach aimed at optimizing the natural protective layer that forms on the enamel.^21^ AEP engineering modifies this process by promoting the early deposition of specific proteins that have high binding ability to hydroxyapatite and are acid-resistant, such as cystatins (e.g., MaquiCPI-3). Following this concept, since the mechanism involves binding of the proteins to hydroxyapatite, pre-treatment enhances the formation of an AEP that is enriched with these protective proteins directly onto the enamel surface, increasing its ability to resist acid attacks and preserve the enamel’s mineral content. Moreover, the duration for AEP formation was adjusted. Although many studies utilize 2 hours for complete pellicle formation, recent research indicates that the pellicle can form in as little as 3 min.^35^ Consequently, we reduced the pellicle formation time to 1 hour, ensuring that this adjustment did not adversely affect the study outcomes.^12^ The erosive challenge was performed with citric acid to replicate the impact of citrus fruits and to address the protocol for extrinsic dental erosion, given that this type of acid is one of the most common in people’s everyday diet.^36^The experiment was limited to three days to simulate the initial stages of dental erosion and assess the early enamel response to erosive conditions. This period aligned with the study’s focus on prevention, as effective intervention is crucial before significant enamel loss occurs.

Regarding the treatments used, the maquiberry-based cystatin MaquiCPI-3 is a novel approach in the study of dental erosion due to its recombinant production using *E. coli*. Recently, this protein was recombinantly expressed and tested at different concentrations in an *in vitro* initial dental erosion protocol.^24^ The optimal concentration of MaquiCPI-3 was found to be 0.5 mg/mL which significantly differed from the 0.1, 0.25, and 1.0 mg/mL concentrations in preliminary experiment.^24^ This finding justifies the 0.5 mg/mL concentration selected in the present study. Additionally, this study showed that 0.5 mg/mL of MaquiCPI-3 provided superior enamel protection compared with 0.1 mg/mL cystatin from sugarcane (CaneCPI-5).^24^ This significant finding, combined with the fact that MaquiCPI-3 has a higher production yield compared with CaneCPI-5, makes it an attractive candidate for developing commercially scalable products.

The likely mechanism by which MaquiCPI-3 protects enamel may resemble that of CaneCPI-5, which showed a strong binding affinity to hydroxyapatite under atomic force microscopy.^20^ This is particularly plausible given that both MaquiCPI-3 and CaneCPI-5 belong to the same cystatin family, suggesting they may function in a comparable manner when applied onto the enamel surface. Additionally, no studies have specifically addressed the chemical forces involved in binding MaquiCPI-3 to hydroxyapatite, as MaquiCPI-3 is a novel protein, and, to the best of our knowledge, no detailed molecular-level analysis has been conducted to date. Future studies will be conducted to address these questions and further investigate the binding mechanisms. Another important mechanism concerns the fact that MaquiCPI-3 enhances AEP protection by increasing the expression of other acid-resistant proteins (e.g., cystatins, statherin, lysozyme, and Proteins S-100) in this integument, as showed by a proteomic study *in vivo* (unpublished data). This mechanism is also similar to CaneCPI-5 in protecting against erosive challenges, as evidenced in *in vitro* and *in vivo* protocols.^15,21^ However, as MaquiCPI-3 in dentistry is its initial stage, future studies are necessary to deep into this hypothesis.

Concerning the inorganic components utilized here, NaF was selected due to its proven efficacy in protecting against oral conditions like dental caries and ETW.^37,38^ The chosen concentration is supported by studies indicating both safety for the oral cavity and maximal protection for the enamel surface. While NaF has been previously tested in combination with recombinant proteins (CaneCPI-5),^12^ Sn^2^ is being evaluated for the first time in this context. This component has also shown positive outcomes in enamel protection against demineralization.^39^ In clinical practice, these two inorganic components are available in a commercial mouthwash aimed at protecting against ETW (Elmex™ Erosion Protection),^40^ which was also used here as a positive control. For both components, the protection mechanism involves forming a protective layer on the enamel surface which enhances resistance to acid dissolution. This layer acts as a physical barrier, reducing enamel solubility and providing additional protection against erosive challenges. Together, these components offer a synergistic effect that strengthens enamel and minimizes dental erosion and ETW.^40^ It is also worth explaining the absence of a group treated only with Sn^2^.

%SMC showed that the absence of a significant difference between NaF and NaF+SnCl solutions can be interpreted in light of the initial erosion model, as the last solution exhibits superior efficacy in scenarios involving more severe erosive challenges.^41^The synergy among all the components within the same solution provided effective enamel protection, achieved through various mechanisms of action both within the acquired pellicle and on the enamel surface (as described in the previous paragraph).^20,21^ However, Maqui+NaF+SnCl did not differ significantly from the NaF+SnCl₂ treatment, indicating that the combination containing only inorganic components also yields favorable results. In the present study, the MaquiCPI-3 group presented a higher %SMC (11.68/4.17) compared with the same group in a previous study (2.90%/3.35).^24^ This discrepancy can be attributed to differences in treatment duration (2 min versus 2 h, respectively) and AEP formation (1 h versus 2 h, respectively).^24^ The extended treatment time in the previous study may have resulted in greater MaquiCPI-3 biding to hydroxyapatite which in turn increased the number of acid-resistant proteins in the AEP, consequently enhancing protection against erosive challenges. Importantly, the treatment duration in the previous study is not compatible with the clinical scenario. Nonetheless, our study found effective protection by MaquiCPI-3 in a shorter and more clinically relevant treatment time.

When comparing the %SMC of the Maqui+NaF+SnCl (6.67/5.58) group, which exhibited the most effective protective effect, with the group containing only MaquiCPI-3 from the previous study (2.90%/3.35),^24^ we note that %SMC becomes more similar between them. This underscores that within a shorter treatment application time (only 2 min), combining MaquiCPI-3 with the inorganic components (F^-^ and Sn^2^) was crucial in maintaining a higher degree of protection compared with isolated MaquiCPI-3,^24^ elucidating the interplay between treatment duration and compositional synergy. Another study used a treatment combining CaneCPI-5 and NaF to prevent initial dental erosion *in vitro* and showed that this combination was more effective in enamel protection (%SMC = 6.8/2.8) when compared with isolated CaneCPI-5 (%SMC = 18.3/2.1%).^12^ Considering this result, we observe that the current combination containing Maqui+NaF+SnCl (6.67/5.58) achieved a similar protection pattern to the previous study. However, the several differences between the studies—type of treatment, number of cycles (5 times versus 3 times), volume of acid (10 mL versus 1 mL), analysis parameters, and brand of the Microhardness tester—make direct comparison difficult.^12^

In analyzing treatment pH, we observed that deionized water (pH 7.0) did not protect the enamel against dental erosion. Despite having a neutral pH, its lack of active components prevents the formation of protective layers on the enamel’s mineral structure. Additionally, its low buffering capacity may facilitate the removal of mineral ions, making the enamel more susceptible to subsequent erosive challenges. Conversely, despite having an acidic pH (~4.43), Elmex™ did not induce dental erosion given the presence of stannous ions (Sn^2^⁺), which rapidly interact with the enamel surface forming a protective layer composed of tin oxides and hydroxides (SnO₂/Sn(OH)₂).^40,41^ This tin-based layer helps to reduce enamel dissolution by providing a physical barrier that limits the interaction between enamel and acid, thereby diminishing the severity of subsequent erosive challenges.

F⁻ plays a crucial role in enhancing enamel resistance to demineralization. Fluoride contributes to fluorapatite (Ca₅(PO₄)₃F) precipitation on the enamel surface, a mineral that is structurally more resistant to acid attack and demineralization compared with natural hydroxyapatite (Ca₅(PO₄)₃OH). This transformation is significant because fluorapatite’s crystal lattice is more stable and less soluble in acidic environments, which provides an additional layer of protection against erosive agents.^41^ In short, the combined effect of tin and fluoride ions helps to fortify the enamel, reducing the potential for mineral loss and improving the long-term durability of the enamel surface against ETW. Although the solution’s pH is acidic, it is not sufficiently low to cause significant enamel dissolution. A similar mechanism may have played out in the NaF and NaF+SnCl groups, both with a pH of 4.50, in which inorganic ions provide protection against erosion.

In contrast, the groups containing MaquiCPI-3 showed a higher pH: MaquiCPI-3 isolated (pH 7.30), Maqui+NaF (pH 7.45), and Maqui+NaF+SnCl (pH 7.33). Considering only the pH of these solutions, their proximity to neutrality (pH 7.30–7.45) favors protection against dental erosion via two main mechanisms: 1) absence of a demineralizing effect, as neutral solutions maintain the enamel’s mineral integrity, unlike acidic solutions which promote hydroxyapatite dissolution; and 2) a favorable environment for remineralization, since a pH close to physiological levels allows saliva to aid in depositing minerals on the enamel surface, increasing its resistance to erosive challenges.^12,21^

%SRI analysis performed by Optipen Reflectometer shows extensive validation in comparison with the recording of dental erosive wear obtained by Basic Erosive Wear Examination (BEWE) in *in vitro* studies.^36^ Moreover, a study revealed that the Reflectometer exhibits a significant correlation between surface microhardness and calcium release following the application of an *in vitro* erosion protocol.^31^ Additionally, a strong correlation has been observed between the Reflectometer and Contact Profilometry analysis, suggesting the potential application of this device for evaluation of *in situ* protocols.^23^ This technique was recently employed for the first time in an *in vivo* protocol, indicating its potential for clinical application and a significant correlation with calcium analysis using the Arsenazo III method.^13^

Our study used, for the first time, the Reflectometer to analyze the protective effect of MaquiCPI-3. %SRI results showed similarity among the groups, ranging from 84.16/9.17 to 90.43/7.16, for treatments with Elmex, NaF+SnCl, MaquiCPI-3, Maqui+NaF, and Maqui+NaF+SnCl. These treatments exhibited significant protection (higher %SRI values) compared with water and NaF. Importantly, the Reflectometer may show interference in reflection measurement due to the presence of proteins from the treatments and AEP.^12^ Even after washing and erosive challenge, proteins can remain attached to the dental enamel surface, increasing %SRI.^22^ Interestingly, we expected NaF to also exhibit a high %SRI, consistent with the degree of protection indicated by %SMC analysis. However, a study found that the F^-^ ion can interact with AEP proteins reducing the protein expression of this integument.^12^ Combining this finding with the potential interference of proteins in reflection, this result likely accounts for the lower %SRI, especially for the group treated with isolated NaF.

Some study limitations should be highlighted, such as the use of a static *in vitro* model which introduces a certain distance from clinical conditions, particularly regarding the dynamics of AEP formation influenced by factors like salivary flow, oral microbiota, and dietary intake.^32^ However, the results obtained offer valuable insights and will pave the way for future research. These endeavors should consider implementing more intricate models, including *in situ* and *in vivo* studies, to validate and extend the present findings and thus ensure a more robust extrapolation of results to clinical practice.

Except for hypothesis number 2, all other null hypotheses were rejected. Specifically, our data did not support the null hypotheses that MaquiCPI-3 does not prevent initial dental erosion *in vitro* (hypothesis 1), or that combining NaF and SnCl_2_ does not have a synergistic effect, either between them or with MaquiCPI-3, to enhance the prevention of initial dental erosion (hypotheses 3 and 4). However, the second hypothesis, which postulated that NaF does not enhance the preventive effect of MaquiCPI-3 against initial dental erosion *in vitro*, was accepted. This suggests that NaF, in combination with MaquiCPI-3, does not show a significant additive effect in preventing initial enamel erosion within the parameters of this study.

In conclusion, the formulations combining organic and inorganic components, such as Maqui+NaF+SnCl, offer superior enamel protection compared with MaquiCPI-3 isolated. The findings are particularly relevant for developing innovative dental products aimed at effectively mitigating ETW.
